# Community-Based Hip Screening for Up to Four-Month-Old Infants and Health Guidance for Their Caregivers in Japan: A Nation-Wide Survey

**DOI:** 10.3390/nursrep13040121

**Published:** 2023-10-11

**Authors:** Kyoko Yoshioka-Maeda, Hiroshige Matsumoto, Asa Inagaki-Asano, Chikako Honda

**Affiliations:** 1 Department of Community Health Nursing, Division of Health Sciences and Nursing, Faculty of Medicine, The University of Tokyo, Tokyo 113-0033, Japan; hiroshige-tky@g.ecc.u-tokyo.ac.jp (H.M.); hchika-tky@g.ecc.u-tokyo.ac.jp (C.H.); 2 Department of Gerontological Home Care and Long-Term Care Nursing, Division of Health Sciences and Nursing, Faculty of Medicine, The University of Tokyo, Tokyo 113-0033, Japan; asainagaki-tky@g.ecc.u-tokyo.ac.jp

**Keywords:** community, developmental dysplasia of the hip, health guidance, hip screening, infant health checkups, newborn home visit, midwives, public health nurses

## Abstract

Early detection of developmental dysplasia of the hip (DDH) in children is crucial. Due to COVID-19, maternal and child health services have been suspended temporarily, increasing the risk of late detection of DDH. This study aimed to reveal Japan’s current situation regarding community hip screening for newborns and infants and to provide health guidance for caregivers regarding DDH. A web-based, nationwide cross-sectional survey was conducted between February and March 2023 (n = 1737). One public health nurse overseeing maternal and child health per municipality responded to the 2022 municipality hip screening system. Among the 436 municipalities that responded (response rate: 25.1%), 97.5% implemented hip screening within 4 months, and approximately 60% performed it during newborn home visits, while only 2.3% conducted hip ultrasound screening. Perfect checking of the risk factors for DDH during newborn home visits and training opportunities for home visitors must be improved. Educational programs regarding DDH for home visitors and caregivers are needed to prevent the late diagnosis of DDH. Furthermore, collaboration between pediatric orthopedic surgeons and nurses is crucial for developing effective community-based hip-screening systems by bridging the evidence and practice gap in the early detection of DDH.

## 1. Introduction

Developmental dysplasia of the hip (DDH) causes adverse health effects, including gait abnormalities, pain, hip osteoarthrosis, and low quality of life [1,2]. Its early detection contributes to effective treatment [2], whereas late-detection cases require follow-up for several decades [3]. Patients with DDH have decreased psychosocial well-being and increased family burden [4,5]. High-risk infants with DDH are female, born in the pelvic position, have a family history of DDH [1,6], were subjected to swaddling [7,8], and were born in winter [9,10]. Although Ortolani and Barlow tests can identify limited abduction and potential DDH, misclassified DDH cases account for 14% of all experts’ physical examinations [11]. Ultrasonography without radiation exposure has been suitable for the early detection of DDH since the 1980s [12,13,14], and a universal ultrasound screening system for the early detection of DDH has been strongly recommended [15]. However, previous studies have mainly focused on diagnoses in hospital settings, and little is known about community-based hip screening. 

The prevalence of DDH in Japan is approximately 0.01% [9]. All local governments conduct home visits and 4-month health checkups, checking the hips of newborns and infants based on the national guidelines [16]. Common checkpoints for assessing suspected DDH cases during newborn home visits and 4-month health checkups are as follows: (1) having an orientation habit and the leg on the opposite side not forming an M-shape; (2) being biologically female; (3) having a positive family history; (4) being born in a breech position; and (5) being born in a cold region or during the cold season (November to March) because caregivers wrap the infant(s) in clothes with the legs outstretched to protect them from cold temperatures [17]. Suspected cases of DDH with limited abduction were immediately referred to medical facilities. In Japan, public health nurses (PHNs) and midwives (MWs) have advised caregivers regarding daily care, including diapers and swaddling to prevent the acquisition of DDH in daily life, since the 1970s. They conduct physical examinations as primary screening during newborn home visits, reducing the number of DDH cases [18,19]. However, most studies were hospital-based and little is known about health guidance for the prevention of DDH in community settings [7,20,21] because child maltreatment is a more popular topic than DDH worldwide [22], as well as in Japan [23]. Nurse experiences with DDH and attention towards assessing related risk factors may be lower than those for child maltreatment. Recognizing the significance of early detection, the national government has implemented a policy to promote the timely identification of DDH cases in community settings since 2021 [24]. Raising nurses’ awareness of DDH is crucial to prevent late DDH cases in community settings.

Japan has two types of home visiting services for developing maternal and child health (MCH): one is newborn home visits focused on enhancing MCH based on the Maternal and Child Health Law of 1965 [25]; the other aims to prevent child maltreatment through PHNs, MWs, and health volunteers based on the Child Welfare Law since 2009 [26]. National guidelines show that municipalities can simultaneously provide two home visiting services [27]. The training staff and management of hip-screening accuracy depend on the local government, yet little is known about whether the staff are nurses, which may be associated with hip-screening in community settings. Additionally, the Japanese Pediatric Orthopaedic Association (JPOA) conducted a nationwide survey in 2013, showing that 15% of the cases were over 1 year old despite having received home visits and infant health checkups in the past [28]. Furthermore, the COVID-19 pandemic has negatively affected the detection of DDH cases [29] because the national government recommended temporarily suspending these services [30]; thus, local governments cannot perfectly conduct newborn and infant home visits [31]. Nurses play a crucial role in providing MCH services in each community, and early detection and intervention in suspected DDH cases would improve the outcomes for affected children. Therefore, this study aimed to examine Japan’s current situation of community hip screening among newborns and infants and provide health guidance for caregivers regarding DDH for early detection and prevention. We hypothesized that nurses do not perfectly implement hip screening and health guidance in community settings.

## 2. Materials and Methods

The present study is a cross-sectional survey of municipalities in Japan and was approved by the Ethics Committee of the University of Tokyo School of Medicine (2022309NI).

### 2.1. Participants and Procedure

In February 2023, the instructions and URL for the web-based survey were mailed to all Japanese municipalities (n = 1737). One PHN per municipality in charge of MCH answered a self-administered questionnaire regarding the 2022 community hip-screening system. For the municipalities where health sections were integrated, the instructions were sent to the association headquarters. The participants responded to the survey in March 2023.

The municipalities that did not respond after 2 weeks received a reminder telephone call. Owing to resource availability, the reminders were incomplete for 66.3% (n = 1151) of the municipalities. All participants gave their informed consent.

### 2.2. Measurements

We developed the list of survey items observed at each checkup based on national guidelines [16] and included the following: name of the municipality; opportunities for infant hip screening (newborn visits, 4-month checkups, other checkup opportunities if applicable, and the target age range for these checkups); five risk factors for DDH observed at each checkup (limited hip abduction, asymmetry of the groin skin folds, female sex, family history of DDH, and breech presentation); birth season; positive Barlow and Ortolani tests, and asymmetrical head turning. Additionally, we included four items regarding newborn home visits: visitor licenses in newborn visits (nursing or other staff); health guidance during newborn visits about the prevention of DDH; content of health guidance (wearing diapers, babywearing, baby carrier, baby clothes, bedding, and swaddling) [17]; and the training of visitors on screening by a municipality. Furthermore, the municipalities were asked whether they used ultrasound for hip screening. 

To reduce nonresponse bias, the municipality’s name was optional. However, since there were only three cases of data with missing municipality names, all data with missing municipality names were excluded from the analysis.

### 2.3. Data Linkage and Coding

The number of births from public data (Ministry of Internal Affairs and Communications) and the population from the census were linked based on the municipality’s name.

We categorized municipalities by their prefectures’ average annual temperature into cold (<15 °C; Hokkaido, Aomori, Iwate, Miyagi, Akita, Yamagata, Fukushima, Tochigi, Niigata, and Nagano; n = 138; range: 10.2–14.9 °C) and warm (≥15 °C; n = 295; range: 15.0–23.6 °C). The incidence of DDH increases during cold weather seasons [9,10]; thus, we hypothesized that municipalities in cold regions are more sensitive to DDH. The threshold was set at 15 °C, which is approximately consistent with prefectures designated as cold/semi-cold regions in the national housing construction standards. We categorized municipalities by population into small (<10,000; n = 95; range: 681–9751), medium (<200,000; n = 291; range: 10,083–199,432), and large (≥200,000; n = 47; range: 202,978–2,732,197).

Hip screening at newborn visits was coded as screening within 2 months. Although visits are legally required to be conducted within the age of 4 months, 73.9% of municipalities do so within the age of 2 months [31]. 

We categorized visitor licenses as nurse only, non-nurse only, or mixed. Nurses included registered nurses, PHNs, and MWs. Non-nurses included qualified nursery teachers, dieticians, welfare commissioners, and community health volunteers.

### 2.4. Analysis

First, we estimated the prevalence of hip screening within 4 months of age based on the number of municipalities and births.

Second, we analyzed the association between hip screening implementation and municipal characteristics (population and climate) using multiple logistic regression analysis. The visitor license was added as an independent variable for screening during the newborn visit. 

Third, we analyzed whether visitor training was associated with observing the recommended risk factors and providing health guidance during visits. 

The significance level was set at two-sided p < 0.05. Due to the exploratory nature of the analysis, no correction was performed for multiple tests.

## 3. Results

**Figure 1:**
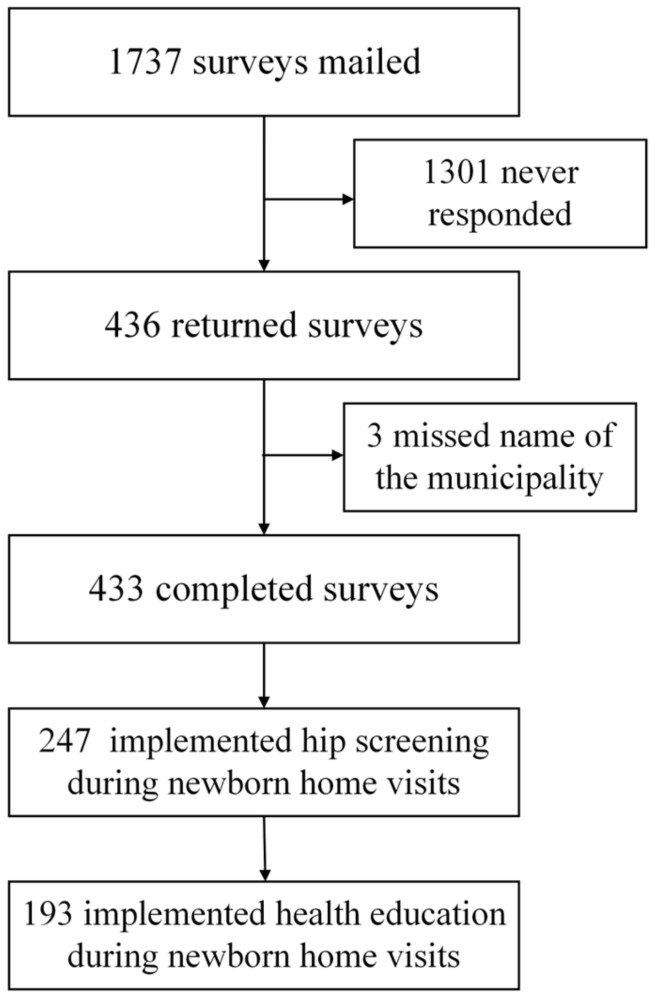
Flow diagram of participants in this study.

A total of 436 municipalities responded; of these, 433 were ultimately included in the analysis (valid response rate: 24.9%). Figure 1 shows the flow diagram of participants in this study.

### 3.1. Current Status of Municipalities’ Hip Screening

Table 1 shows the status of hip screening in the municipalities. In this sample, 422 municipalities (97.5%) implemented hip screening within 4 months and 262 (60.5%) within 2 months. With regard to screening opportunities within 2 months, 247 municipalities conducted screening during newborn visits. An estimate based on the number of births showed that 97.1% and 53.9% of Japanese infants underwent hip screening within 4 and 2 months, respectively. Ten municipalities (2.3%) conducted ultrasound screening.

Table 2 shows the association between the implementation of hip screening and municipal characteristics. Significantly fewer municipalities underwent hip screening within 4 months, within 2 months, and at newborn visits in colder regions than in other regions (adjusted odds ratios [AOR] = 0.17, 0.51, and 0.33, respectively). Municipalities with more than 200,000 residents were significantly less likely than those with less than 10,000 residents to undergo hip screening within 2 months and during newborn visits (AOR = 0.32 and 0.40, respectively). Additionally, we found a significant association between newborn visits by nurses and hip screenings. Screening during newborn visits was performed in 61.4%, 22.7% and 46.4% of municipalities where visitors were nurses only, non-nurses-only (AOR = 0.14), and a mix of nurses and non-nurses (AOR = 0.42), respectively.

### 3.2. Observed Risk Factors, Health Guidance, and Staff Training for Newborn Visits

Table 3 provides details of the municipalities that implemented hip screening during newborn home visits (*n* = 247), with 29.1% (*n* = 72) thoroughly observing the five recommended five risk factors (i.e., limited hip abduction, asymmetry of the groin skin folds, female sex, family history of DDH, and breech presentation). Health guidance for caregivers to prevent DDH was provided during newborn visits in 193 (78.1%) municipalities. Sixty-two municipalities (25.1%) provided DDH prevention training for all visitors. Except for the association between training for visitors and the municipality population, we found no significant associations between municipality characteristics and the observation of risk factors, health guidance, or training.

Table 4 shows the results of the multiple logistic regression analysis with risk factor observation and health guidance as the outcomes. After adjusting for population, cold climate, and visitor type, visitor training was found to be significantly associated with the observation of recommended risk factors (AOR = 4.19) and implementation of health guidance (AOR = 2.60). 

Among the municipalities screened during newborn visits (*n* = 247), limited hip abduction (93.1%) was the most frequently observed risk factor (Table 5). A family history of DDH (41.7%), breech presentation (44.5%), Barlow and Ortolani tests (48.2%), birth season (23.1%), and asymmetrical head turning (49.4%) were observed in less than half of the municipalities. The most common health guidance topics for caregivers were baby-wearing and diapers (Table 5).

## 4. Discussion

This is the first study on community-based hip screening for and health guidance on DDH for infants’ caregivers in Japan. While 97.5% of the responding local governments performed hip screenings at any moment during the checkups in the first four months, approximately half of the municipalities conducted hip screenings during newborn and infant home visits. We found that 2.3% of municipalities in Japan conducted hip ultrasonography screening. Although there is a controversial discussion surrounding whether or not universal or selective hip screening is used for the early detection of DDH [15,32], ultrasound hip screening is vital to detect DDH without radiation exposure [1,12]. Our findings suggest that Japan should develop a community-based hip-screening system using ultrasonography. Additionally, existing newborn and infant home visits would be a good opportunity for nurses to examine and support newborns and infants in their healthy hip development. This concept is similar to universal health coverage, which promotes community health and which nurses play an essential role in strengthening [33]. Nurses have used point-of-care ultrasonography (POCUS) to improve the quality of care in hospitals [34,35]. With the application of POCUS, PHNs and MWs can conduct ultrasonography for hip screening during newborn and infant home visits to detect DDH. Therefore, an educational program needs to be developed for them. 

In our results, approximately 30% of the participants assessed five risk factors for DDH, which were limited—only a quarter of the municipalities provided training for all visitors. Training visitors was positively associated with confirming the well-known risk factors of DDH [1] and providing health guidance to infants’ caregivers after adjusting for population level, climate, and visitor type. Owing to limited budgets, in addition to nurses, health volunteers support maternal and child health care in the community [36,37]. The healthy development of children and prevention of child maltreatment have become worldwide goals [38]. DDH is also a significant global issue because it is associated with walking, which is directly related to children’s quality of life, and increases their family burden [1,5]. Risk factor screening is crucial for the early detection of DDH [39]. However, our results suggest a gap between research and practice. Further research is required to develop questionnaires and training materials that can quickly identify the risk factors for DDH in health volunteers. In addition, more information is needed regarding the assessment and prevention of DDH in nursing practice [40]. Future studies are required to identify the status of nurses’ DDH knowledge and assessment skills, including their undergraduate education and skill development. 

We found that municipalities did not provide adequate health guidance for preventing DDH during newborn and infant home visits, and less than half advised caregivers on bedding and swaddling. Historically, health guidance for caregivers’ daily care of newborns and infants has helped prevent DDH [18,19]. Worldwide, tight swaddling is commonly used by caregivers, including nurses, to calm newborns and infants and to protect them from cold temperatures [20,40]. Safe hip positioning and swaddling with natural flexion of the leg reduce the risk of DDH [21,22]. Although the prevalence of DDH varies in each country [1], nurses need to support caregivers in learning how to prevent DDH through daily care. Attractive educational materials for caregivers are needed to help prevent potential DDH cases and to promote a hip-safe community.

This study has several limitations. First, the response rate was low, with many missing data points. Due to a limited schedule, we surveyed at the end of the fiscal year, coinciding with local government personnel changes. In addition, local governments in Japan had to respond to the COVID-19 pandemic. Second, a single respondent in charge of MCH represented the organization and may have only partially reflected its views. The municipalities that responded might have had keen interest in DDH; thus, the implementation of DDH screening may have been overestimated. We called 60% of communities as a reminder, but we could not cover all municipalities to reduce sampling bias; therefore, the generalizability of this study’s results is limited. Third, we could not identify the training procedures for nurses and non-nurses in each municipality. Fourth, the differing nature of PHNs and MWs may have influenced the test’s inter- and intra-rater reliability, affecting the quality of hip screening.

Nevertheless, this study provides new evidence on the status of primary hip screening and health guidance for caregivers regarding DDH in community settings. Our results suggest that the existing MCH care systems would be equivalent to universal screening and require improved accuracy and performance. Additionally, developing educational programs for home visitors and caregivers is necessary to enhance effective community-based hip screening for the early detection and prevention of DDH.

## 5. Conclusions

This nationwide cross-sectional study revealed that, although most local governments conducted hip screening during the 4-month infant checkup, approximately 60% performed it during newborn home visits. A perfect assessment of the risk factors for DDH during newborn home visits and training opportunities for home visitors were lacking, and community-based ultrasound hip screening has only recently begun in Japan. To prevent the late diagnosis of DDH, educational programs for nurses, health volunteers and caregivers are needed. Collaborating with pediatric orthopedic surgeons and nurses is crucial for developing effective community-based ultrasound hip screening systems by bridging the evidence and practice gap to prevent the late diagnosis of DDH cases.

## Figures and Tables

**Table 1:** Status of municipalities’ hip screening (n = 433).

			Municipality, *n*	(%)	(95% CI)	Births, *n*	(%)
Hip screening							
	Within 4 months	Absent	11	(2.5)	(1.1–4.0)	8624	(2.9)
		Present	422	(97.5)	(96.0–98.9)	291,872	(97.1)
	Within 2 months	Absent	171	(39.5)	(34.9–44.1)	138,434	(46.1)
		Present	262	(60.5)	(55.9–65.1)	162,062	(53.9)
	At newborn visits	Absent	186	(43.0)	(38.3–47.6)	141,519	(47.1)
		Present	247	(57.0)	(52.4–61.7)	158,977	(52.9)
	Using ultrasound *	Absent	397	(91.7)	(89.1–94.3)	256,658	(85.4)
		Present	10	(2.3)	(0.9–3.7)	6371	(2.1)
Total			433	(100.0)		300,496	(100.0)

* Twenty-six municipalities have missing values concerning the use of ultrasound.

**Table 2:** The association between hip screening implementation and municipality characteristics (*n* = 433).

		Hip Screening																	
		Within Four Months						Within Two Months						At Newborn Visit					
		Absent		Present				Absent		Present				Absent		Present			
		*n*	(%)	*n*	(%)	AOR	*p*	*n*	(%)	*n*	(%)	AOR	*p*	*n*	(%)	*n*	(%)	AOR	*p*
Regional climate																			
	Warm	4	(1.4)	291	(98.6)			105	(35.6)	190	(64.4)			112	(38.0)	183	(62.0)		
	Cold	7	(5.1)	131	(94.9)	0.17	0.007	66	(47.8)	72	(52.2)	0.51	0.003	74	(53.6)	64	(46.4)	0.33	<0.001
Population																			
	<10,000	1	(1.1)	94	(98.9)			36	(37.9)	59	(62.1)			39	(41.1)	56	(58.9)		
	<200,000	8	(2.7)	283	(97.3)	0.19	0.130	108	(37.1)	183	(62.9)	0.78	0.359	120	(41.2)	171	(58.8)	0.75	0.296
	≥200,000	2	(4.3)	45	(95.7)	0.10	0.067	27	(57.4)	20	(42.6)	0.32	0.003	27	(57.4)	20	(42.6)	0.40	0.020
Newborn visitor																			
	Nurses only													132	(38.6)	210	(61.4)		
	Non-nurses only													17	(77.3)	5	(22.7)	0.14	0.000
	Mixed													37	(53.6)	32	(46.4)	0.42	0.002
Total		11	(2.5)	422	(97.5)			171	(39.5)	262	(60.5)			186	(43.0)	247	(57.0)		

Note. AOR: adjusted odds ratio, Cold: municipalities located in prefectures with an average annual temperature below 15 °C.

**Table 3:** Observation of risk factors, health guidance, and visitor training for newborn visits (*n* = 247).

		Observation of the Recommended Five Risk Factors						Health Guidance for Caregivers						Training for All Visitors					
		Not Complete		Complete				Absent		Present				Absent		Present			
		*n*	(%)	*n*	(%)	AOR	*p*	*n*	(%)	*n*	(%)	AOR	*p*	*n*	(%)	*n*	(%)	AOR	*p*
Regional climate																			
	Warm	136	(74.3)	47	(25.7)			44	(24.0)	139	(76.0)			138	(75.4)	45	(24.6)		
	Cold	39	(60.9)	25	(39.1)	1.80	0.099	10	(15.6)	54	(84.4)	2.20	0.070	47	(73.4)	17	(26.6)	1.60	0.212
Population																			
	<10,000	34	(60.7)	22	(39.3)			14	(25.0)	42	(75.0)			46	(82.1)	10	(17.9)		
	<200,000	131	(76.6)	40	(23.4)	0.59	0.144	36	(21.1)	135	(78.9)	1.74	0.173	127	(74.3)	44	(25.7)	1.94	0.126
	≥200,000	10	(50.0)	10	(50.0)	2.11	0.193	4	(20.0)	16	(80.0)	2.12	0.271	12	(60.0)	8	(40.0)	3.77	0.034
Newborn visitor																			
	Nurses only	151	(71.9)	59	(28.1)			46	(21.9)	164	(78.1)			159	(75.7)	51	(24.3)		
	Non-nurses only	2	(40.0)	3	(60.0)	4.87	0.095	0	(0.0)	5	(100.0)	465,877,307.66	0.999	4	(80.0)	1	(20.0)	0.85	0.883
	Mixed	22	(68.8)	10	(31.3)	1.24	0.626	8	(25.0)	24	(75.0)	0.88	0.773	22	(68.8)	10	(31.3)	1.33	0.505
Total		175	(70.9)	72	(29.1)			54	(21.9)	193	(78.1)			185	(74.9)	62	(25.1)		

Note. AOR: adjusted odds ratio, Cold: municipalities located in prefectures with an average annual temperature below 15 °C.

**Table 4:** The association between observation of risk factors, health guidance, and visitor training for newborn visits (*n* = 247).

		Observation of the Recommended Five Risk Factors						Health Guidance for Caregivers					
		Not Complete		Complete				Absent		Present			
		*n*	(%)	*n*	(%)	AOR	*p*	*n*	(%)	*n*	(%)	AOR	*p*
Training for all visitors													
	Absent	145	(78.4)	40	(21.6)			47	(25.4)	138	(74.6)		
	Present	30	(48.4)	32	(51.6)	4.19	<0.001	7	(11.3)	55	(88.7)	2.60	0.031

Note. AOR: adjusted odds ratio, adjusted for population, cold climate, and visitor types.

**Table 5:** The contents of observed risk factors and health guidance.

**Observed Risk Factors (*n* = 247)**	**Not Observe**		**Observed**	
	***n***	**(%)**	***n***	**(%)**
Limited hip abduction *	17	(6.9)	230	(93.1)
Asymmetry of the groin skin folds *	37	(15.0)	210	(85.0)
Female sex *	119	(48.2)	128	(51.8)
Family history of DDH *	144	(58.3)	103	(41.7)
Breech presentation *	137	(55.5)	110	(44.5)
Barlow and Ortolani tests	128	(51.8)	119	(48.2)
Birth season	190	(76.9)	57	(23.1)
Asymmetrical head turning	125	(50.6)	122	(49.4)
**Health guidance (*n* = 193)**	**Absent**		**Present**	
	***n***	**(%)**	***n***	**(%)**
Wearing diapers	41	(21.2)	152	(78.8)
Babywearing	12	(6.2)	181	(93.8)
Baby carrier	91	(47.2)	102	(52.8)
Baby clothes	79	(40.9)	114	(59.1)
Bedding	100	(51.8)	93	(48.2)
Swaddling	111	(57.5)	82	(42.5)

* The recommended five risk factors in Japan’s national guideline.

## Data Availability

The data presented in this study are available on request from the corresponding author. The data are not publicly available due to protect the privacy of each local government.

## Guidelines and Standards Statement

This manuscript was drafted against the STROBE Statement for cross-sectional studies. A complete list of reporting guidelines can be accessed via the STROBE checklists: https://www.strobe-statement.org/checklists/ (accessed on 19 June 2023).
